# Effects of an 8-month exercise intervention on physical capacity, NT-proBNP, physical activity levels and quality of life data in patients with pulmonary arterial hypertension by NYHA class

**DOI:** 10.1016/j.dib.2017.03.022

**Published:** 2017-03-18

**Authors:** Alejandro Santos-Lozano, Paz Sanz-Ayan, Laura González-Saiz, Carlos A. Quezada-Loaiza, Carmen Fiuza-Luces, Angela Flox-Camacho, Diego Munguía-Izquierdo, Alfredo Santalla, María Morán, Pilar Escribano-Subías, Alejandro Lucia

**Affiliations:** a Research Institute Hospital 12 de Octubre (׳i+12׳), Madrid, Spain; b GIDFYS, European University Miguel de Cervantes, Valladolid, Spain; c Department of Rehabilitation, Hospital Universitario 12 de Octubre, Madrid, Spain; d European University, Madrid, Spain; e Pulmonary Hypertension Unit, Hospital Universitario 12 de Octubre, Madrid, Spain; f Department of Sports and Computer Science, Pablo de Olavide University, Sevilla, Spain

**Keywords:** Pulmonary arterial hypertension, Physical activity levels, Quality of life, Physical intervention

## Abstract

This article provides descriptive detailed (pre and post) values of physical capacity variables, NT-proBNP, physical activity levels and quality of life in patients with pulmonary arterial hypertension (PH) (both, intervention and control group) by New York Heart Association (NYHA) class before and after an 8-month exercise intervention. The data are supplemental to our original Randomized Controlled Trial (RCT) entitled “Benefits of skeletal-muscle exercise training in pulmonary arterial hypertension: The WHOLEi+12 trial” (L. González-Saiz, C. Fiuza-Luces, F. Sanchis-Gomar, A. Santos-Lozano, C.A. Quezada-Loaiza, A. Flox-Camacho, D. Munguía-Izquierdo, I. Ara, A. Santalla, M. Morán, P. Sanz-Ayan, P. Escribano-Subías, A. Lucia A, 2017) [1].

**Specifications Table**

**Table:** 

Subject area	*Medicine*
More specific subject area	*Cardiology*
Type of data	*Table*
How data was acquired	*Functional clinical assessment of the patients and sample blood analysis*
Data format	*Descriptive data: mean*±*SD*
Experimental factors	*Data of the patients with pulmonary arterial hypertension by New York Heart Association class*
Experimental features	*Randomized Controlled Trial*
Data source location	*Madrid, Spain*
Data accessibility	*The data are accessible within the article*

**Value of the data**

• Data presented here provide the descriptive overview of the PH patients by NYHA class involved in an exercise RCT.
• We describe the changes in physical capacity-related primary outcomes (peak watts in leg and bench press) and the changes in physical capacity-related secondary outcomes [maximal inspiratory pressure (PImax), 6-minute walking distance (6MWD), and performance time in the 5-repetition sit-to-stand (5-STS) test] of the PAH patients by NYHA class involved in the RCT (control vs. intervention).
• We describe values of physical activity (PA) levels (min/day in inactivity and moderate-vigorous PA) of PH patients by NYHA class involved in the RCT (control vs. intervention).
• We show the values of health related quality of life [HRQoL, (mental and physical components of SF-36 questionnaire)] of PH patients by NYHA class involved in the RCT (control vs. intervention).
• This data could be useful to physicians to make comparisons with other cohort datasets and so to contribute to the knowledge of the PA effect in PH patients by NYHA class.

## 1 Data

Descriptive values of physical capacity-related variables, NT-ProBNP, cardiopulmonary exercise capacity, PA levels and HRQoL of PAH patients involved in the RCT [2] (ClinicalTrials.gov ID: NCT02288442) conducted from January 2015 to June 2016 at the Hospital 12 de Octubre (Madrid, Spain) following the *Consolidated Standards of Reporting Trials* (CONSORT) guidelines [3] are shown in Table 1.

## 2 Experimental design, materials and methods

20 PH patients were involved in the intervention group of the RCT and 20 PH patients in the control group. The RCT intervention lasted 8 weeks and included 3 main components: aerobic and muscle resistance, and specific inspiratory muscle training. All sessions were supervised by experienced fitness instructors. Data outcome (by order of aquisition) were: i) blood sampling for NT-proBNP determination, PImax, 6MWD, muscle power, 5-STS, and distribution of HRQoL questionnaire (1st day); ii) cardiopulmonary exercise testing (after 2–3 days to allow recovery from power testing); and iii) seven days of accelerometry recording for objective PA determination. Please see Gonzalez-Saiz et al. (2017) [1] for detailed information.

## Figures and Tables

**Table 1:** Results of the primary and secondary end points by group and NYHA class.

		**NYHA Class I**			**NYHA Class I-II**			**NYHA Class II**			**NYHA Class II-III**			**NYHA Class III**		
**End points**	**Group**	***N* with initial data**	**Baseline**	**Post**	***N* with initial data**	**Baseline**	**Post**	***N* with initial data**	**Baseline**	**Post-**	***N* with initial data**	**Baseline**	**Post**	***N* with initial data**	**Baseline**	**Post**

**Primary**																
Leg press	Control	6	551±285	583±200	2	413±178	347	9	308±138	323±130	–	–	–	2	206±136	138
(peak watts)	Exercise	3	372±96	471±130	2	451±437	666±584	11	307±234	507±297	4	310±221	470±241	–	–	–
Bench press	Control	5	297±130	325±135	2	101±28	93	10	119±79	121±59	–	–	–	2	74±34	65
(peak watts)	Exercise	3	151±113	187±99	2	175±174	230±212	11	113±76	173±118	4	109±83	162±72	–	–	–
**Secondary**																
*Blood variables*																
NT-proBNP	Control	4	63±39	35±112	2	141±55	112	8	270±277	427±560	–	–	–	2	344±337	598
(pg/mL)	Exercise	3	560±327	710±558	2	256±193	193±117	11	235±210	210±177	4	264 ±205	263±165	–	–	–
**CET**																
VO_2peak_	Control	6	27.3±5.4	27.9±5.9	2	18.2±0.3	19	9	16.5±2.8	15.5±3.12	–	–	–	2	13±1.4	14
(mL/kg/min)	Exercise	3	16.7±0.56	19±1	2	17.3±3.7	21.3±1.7	11	15.2±3.4	17.3±2.9	4	15.2±4.6	17.4±4.2	–	–	–
P_ET_CO_2_@AT (mmHg)	Control	6	28±3	30±2	2	26±1	27	8	32±6	30±7	–	–	–	2	21±5	22
	Exercise	3	24±6	24±6	2	25±2	23±3	11	28±7	27±7	4	27±4	26±7	–	–	–
P_ET_O_2_@AT	Control	6	113±4	111±2	2	115±2	113	8	110±5	111±5	–	–	–	2	119±4	118
(mmHg)	Exercise	3	116±6	116±3	2	115±5	118±3	11	113±6	115±6	4	114±6	113±8	–	–	–
V_E_/VCO_2_@AT	Control	6	42±7	39±4	2	42±5	37±4	8	38±7	39±9	–	–	–	2	52±16	52
	Exercise	3	45±11	45±12	2	43±2	47±5	11	41±11	44±13	4	43±7	43±10	–	–	
V_E_/VO_2_@AT	Control	6	45±9	43±4	2	45±2	43	8	40±6	43±10	–	–	–	2	58±19	62
	Exercise	3	52±16	54±16	2	46±5	53±7	11	46±12	49±13	4	48±11	48±17	–		
**Other physical capacity variables**																
PI_max_	Control	6	97±32	100±34	2	66±10	60	9	68±24	69±26	–	–	–	2	38±27	83
(cmH_2_O)	Exercise	3	62±33	102±41	2	74±66	105±90	11	81±39	110±25	4	70±26	117±16	–	–	–
6MWD	Control	6	639±97	620±154	2	579±43	549	10	497±75	491±45	–	–	–	2	480±17	492
(meters)	Exercise	3	549±36	572±15	2	504±77	549±43	11	495±76	512±74	4	475±73	517±90	–	–	–
5-STS (performance time, seconds**)	Control	6	6.2±1.2	5.9±1	2	5.1±1	5.2±1	10	7.8±1.5	7.6±1.2	–	–	–	2	6.8	8.1
	Exercise	3	6.5±2	5.8±2	2	6.5±2	5.5±1	11	8±1.5	6.1±1.3	3	7.3±1	6.1±1	–	–	–
																
**PA levels**																
Inactivity time (min/day)	Control	3	531±154	573±100	1	605	674	7	549±109	581±76	–	–	–	2	604±26	668±61
	Exercise	3	562±40	583±53	2	621±136	409±12	6	554±93	536±85	4	573±148	630±101	–	–	–
MVPA	Control	3	42±29	26±20	1	42	49	6	45±26	32±21	–	–	–	2	20±22	16±10
(min/day)	Exercise	3	58±12	13±11	2	42±22	49±5	6	28±19	42±18	4	44±28	34±7	–	–	–
**HRQoL (SF-36)**																
Mental	Control	3	56±6	53±7	1	58	62	7	47±12	53±7	–	–	–	2	49±12	38
component	Exercise	3	49±12	38	2	54±4	59±5	10	45±10	53±10	4	56±6	52±9	–	–	–
Physical component	Control	3	44±4	49±6	1	50	46	8	39±7	43±7	–	–	–	2	35±1	41
	Exercise	3	39±9	42±8	2	45±0.2	50±1	10	37±7	42±6	4	30±6	33±9	–	–	–

Data are mean±SD. Abbreviations: 6MWD, 6-minute walking distance; 5-STS, 5-repetition sit-to-stand; CET, cardiopulmonary exercise testing: HRQoL, health-related quality of life; MVPA, moderate-vigorous physical activity; NT-proBNP (pg/mL), N-terminal pro-B-type-natriuretic peptide; P_ET_CO_2_@AT, end-tidal pressure of carbon dioxide at the anaerobic threshold; P_ET_O_2_@AT, end-tidal pressure of oxygen at the anaerobic threshold; PI_max_, maximal inspiratory pressure; SF-36, Short Form-36 Item Health Survey; V_E_/VCO_2_@AT, ventilatory equivalent for carbon dioxide at the anaerobic threshold; V_E_/VO_2_@AT, ventilatory equivalent for oxygen at the anaerobic threshold; VO_2peak_, peak oxygen uptake.

## References

[1] L. González-Saiz, C. Fiuza-Luces, F. Sanchis-Gomar, A. Santos-Lozano, C. Quezada-Loaiza, A. Flox-Camacho, D. Munguía-Izquierdo, I. Ara, A. Santalla, M. Morán, P. Sanz-Ayan, P. Escribano-Subías, A. Lucia Benefits of skeletal-muscle exercise training in pulmonary arterial hypertension: The WHOLEi+12 trial. Int. J. Cardiol., 231, 2017, 277–283. 10.1016/j.ijcard.2016.12.026 28189191

[2] Sanchis-Gomar F., Gonzalez-Saiz L., Sanz-Ayan P., Fiuza-Luces C., Quezada-Loaiza C.A., Flox-Camacho A. (2015). Rationale and design of a randomized controlled trial evaluating whole muscle exercise training effects in outpatients with pulmonary arterial hypertension (WHOLEi+12). Cardiovasc. Drugs. Ther..

[3] Moher D., Hopewell S., Schulz K.F., Montori V., Gøtzsche P.C., Devereaux P.J., Elbourne D., Egger M., Altman D.G. (2012). CONSORT. CONSORT 2010 explanation and elaboration: updated guidelines for reporting parallel group randomised trials. Int. J. Cardiol..

