# Supplementary material for: Effects of an 8-month exercise intervention on physical capacity, NT-proBNP, physical activity levels and quality of life data in patients with pulmonary arterial hypertension by NYHA class
Source: Data Brief. 2017 Mar 18;12:37–41. doi: 10.1016/j.dib.2017.03.022 (PMC5367801; doi:10.1016/j.dib.2017.03.022)
Supplement: Supplementary file 1 — Supplementary material [file mmc1.docx]

**CONFLICT OF INTEREST**

The authors declare no conflict of interest.
